# Impact of genotypic errors with equal and unequal family contribution on accuracy of genomic prediction in aquaculture using simulation

**DOI:** 10.1038/s41598-021-97873-5

**Published:** 2021-09-15

**Authors:** N. Khalilisamani, P. C. Thomson, H. W. Raadsma, M. S. Khatkar

**Affiliations:** 1 ARC Research Hub for Advanced Prawn Breeding, James Cook University, Townsville, QLD 4811 Australia; 2 Sydney School of Veterinary Science, Faculty of Science, The University of Sydney, Camden, NSW 2570 Australia; 3 School of Life and Environmental Sciences, Faculty of Science, The University of Sydney, Camden, NSW 2570 Australia

**Keywords:** Animal breeding, Genomics

## Abstract

Genotypic errors, conflict between recorded genotype and the true genotype, can lead to false or biased population genetic parameters. Here, the effect of genotypic errors on accuracy of genomic predictions and genomic relationship matrix are investigated using a simulation study based on population and genomic structure comparable to black tiger prawn, *Penaeus monodon*. Fifty full-sib families across five generations with phenotypic and genotypic information on 53 K SNPs were simulated. Ten replicates of different scenarios with three heritability estimates, equal and unequal family contributions were generated. Within each scenario, four SNP densities and three genotypic error rates in each SNP density were implemented. Results showed that family contribution did not have a substantial impact on accuracy of predictions across different datasets. In the absence of genotypic errors, 3 K SNP density was found to be efficient in estimating the accuracy, whilst increasing the SNP density from 3 to 20 K resulted in a marginal increase in accuracy of genomic predictions using the current population and genomic parameters. In addition, results showed that the presence of even 10% errors in a 10 and 20 K SNP panel might not have a severe impact on accuracy of predictions. However, below 10 K marker density, even a 5% error can result in lower accuracy of predictions.

## Introduction

Advanced animal breeding utilizes tools of reproductive biology, molecular genetics, statistics and computer programming in order to optimize the breeding design and enhance the desired commercial traits^1^. The ultimate goal of such programs is to achieve high-production efficiency through long-term genetic gain whilst successfully managing the rate of inbreeding using information on pedigree, genotypes, or haplotypes^2–5^. The application of genomic information in breeding design, i.e. genomic selection (GS), has been widely adopted for enhancing commercial traits in animal breeding^6^. In aquaculture for example, GS has been shown to predict breeding values (BV) more accurately for growth traits in Atlantic salmon^7^, common carp^8^, Nile tilapia^9^, channel catfish^10^, large yellow croaker^11^, yellowtail kingfish^12^, yellow drum^13^, Pacific oyster^14^, scallop^15,16^, whiteleg shrimp^17^ and banana shrimp^18^ compared to pedigree-based BV predictions and has recently been reviewed for applications in aquaculture by Zenger et al.^19^. GS uses the information obtained from genotypic markers to improve the accuracy of BVs. Estimated BVs inferred from molecular markers are termed genomic estimated breeding values (GEBVs), and can be used to accurately select the high-performing candidates for optimising breeding program^20,21^.

Among the many types of genomic markers, single nucleotide polymorphisms (SNPs) have primarily been used in GS due to four reasons, namely: (1) SNPs are relatively inexpensive to process as genetic markers; (2) SNPs are highly abundant and distributed across the genome; (3) they can capture a large proportion of the genetic variation through linkage disequilibrium (LD); and (4) their inheritance to the next generation is more stable than other markers, allowing multi-generation tracking^22–24^. Different sequencing platforms are available for detecting and analysing SNPs. This includes (1) genotype-by-sequencing (GBS); (2) fixed high-density SNP arrays; and (3) low-density SNP panels. GBS does not necessarily provide genotypic data for all the detected SNPs in the population^25–27^. There are situations in quantitative genetics where the analysis requires reliable allelic information of the same loci across all the samples. For most applications, fixed arrays (> 40–50 K) are sufficient to capture genome-wide information. However, high-density SNP chips are generally expensive. This limits their use in routine agricultural applications. Hence, application of low-density SNP panels combined with imputation methods to generate higher density SNP genotypes, usually based on a reference panel^25^, can be a more cost-effective alternative. This approach has recently been extensively used in GS^28–32^.

Despite advancements in sequencing technologies, application of any of these three platforms could create errors in genotypic data. These errors occur mainly due to the structure of sequencing process and human–environmental factors. Genotypic errors might be inherent to the design of the study, e.g., failure of sequencing which can result in detection of null alleles or allelic dropout^33,34^. In addition, errors in genotypic data could also be generated due to human mistake in the laboratory environment, e.g., contamination of DNA samples^35,36^. Some of the errors can be detected by analysing deviation from Hardy–Weinberg equilibrium (HWE)^34,37^, LD analysis within populations^38^, pedigree reconstruction^34,37–40^ and comparison with high-quality reference genotypes^33,40^. Once detected, erroneous genotypes could be filtered out or corrected. To correct the error, one solution could be re-genotyping of a sufficiently large number of individuals and compare it with the first set of genotyped samples, although this is a labour-intensive and expensive practice^34^. However, if the errors are known, imputation methods, based on, for example, application of maximum likelihood or Bayesian algorithms can be applied to estimate most probable genotypes^40,41^.

Population genetic studies have shown that genotypic errors could reduce the power of gene mapping and association studies^36,38,42–44^, bias the estimation of frequency of haplotypes and genotypes^42,44,45^, degrade the accuracy of parentage assignment via false exclusion of parents from assignment^33,34,46,47^, return a false identification of individuals^47^, misrepresent the population structure^47^, underestimate the heterozygosity, departure from HWE and inbreeding coefficients^41,47^. To the best of our knowledge, the only relevant study in simulation breeding design using GS in aquaculture was conducted recently on the effect of genotypic error on the accuracy of genomic prediction^48^. Using population and genome structure from empirical breeding design of rainbow trout *Oncorhynchus mykiss*, they showed that implementing up to 10% error did not significantly impact the accuracy of genomic estimated breeding values (GEBV) across three heritabilities (*h*^2^: 0.1, 0.2, 0.4).

The objective of the current study was to evaluate the effect of genotypic error, family contribution, SNP density and heritability on the accuracy of genomic prediction and medium-term selection response. The range of these parameters investigated was chosen to mirror those observed in the black tiger prawn, *Penaeus monodon*.

## Methods

### Simulation procedure

#### Generating populations

The QMSim software^49^ was used to simulate pedigree with its associated SNP genotypes and phenotypic values. Firstly, 400 historic generations with a constant population size of 1000 in each generation were simulated. In each historical generation, 500 males and 500 females were produced with random selection and random mating. From the last historic population, 50 males and 50 females were randomly selected to form a base population (G0). Then, these 50 males and 50 females of G0 were used as parents and mated randomly to generate 50 full-sib families in the first generation (G01). Within each generation, from G01 to G05, 50 sires and 50 dams across families with the highest estimated breeding values (EBVs), calculated within QMSim, were selected to generate 50 full-sib families in the next generation. Following this breeding design, two broad scenarios based on the size of families were considered:

**Scenario 1 (S1)**: equal family contribution with 100 progeny per family where the probability of producing male and female progeny was 0.5. Fifty families with 100 progeny per family produced 5000 individuals per generation.

**Scenario 2 (S2)**: unequal family contribution was generated with family sizes of 5, 25, 50, 75, 100, 125, 150, 175 and 200 progeny with contribution probability of 5, 10, 12, 14, 18, 14, 12, 10 and 5%, respectively. This means that on average 5% of families were generated with 5 progeny, 10% with 25 progeny, and 12% with 50 progeny, etc. This was to keep the population size per generation as close as possible to number of individuals per generation in **S1** (5000). In addition, the allocation of the number of progeny and their respective distribution was based on the study of maintaining the genetic diversity in *P. monodon* carried out by Foote, et al.^50^. They found that the highest contribution of a single family for *P. monodon* bred in captivity was 18%.

Next, each scenario was divided in three datasets referring to the low, medium, and high heritability traits. In the first dataset, a trait was simulated with medium heritability (0.3), standardized mean of 0 and phenotypic variance of 1. Dominance and epistasis effects were considered absent. The phenotypic values were obtained by summing the random error, the polygenic effect, and the sum of the quantitative trait loci (QTL) effects generated by QMSim software. To allow both QTL and polygenic effects to contribute to variation of the trait, the combined effect of all QTLs were sampled from a normal distribution with mean (μ) of 0 and additive genetic variance (${\sigma }_{a}^{2}$) of 0.2, allowing a third of the variance (0.1) to be attributed to the polygenic effect. The datasets for traits with low (0.05) and high (0.5) heritability were also generated for both S1 and S2 scenarios with additive variance (${\sigma }_{a}^{2}$) of 0.03 and 0.3, respectively. Every dataset was simulated in ten independent replicates, extending the number of datasets to 60 (2 scenarios (family contributions) × 3 trait heritability × 10 replicates). The summary of main scenarios and number of datasets is provided in Table 1.

**Table 1:** The composition of main scenarios and datasets generated for investigating the effect of genotypic error on accuracy of genomic predictions.

Scenario	Family contribution	Heritability (*h*^2^)				
		0.05	0.3	0.5	No. of replicates	No. of datasets
**S1**	Equal				10	30
**S2**	Unequal				10	30
**Total**	–	–	–	–	–	60

#### Genome structure

For each replicate of the generated pedigree within each of the simulation scenarios, a genome was simulated using 44 chromosomes, a number close to the genome structure of *P. monodon*^51^, however, the length of each chromosome was kept as 100 cM for keeping the design simple to implement. On average, 1200 SNPs and 85 QTLs were generated per chromosome. The allocation of 1200 SNP per chromosome was to make sure that every scenario has at least 20 K informative SNPs in Generation 5. The positioning of SNPs and QTLs was random within each chromosome. This allowed the simulation of 52,800 biallelic SNPs and 3740 biallelic QTL genotypes across whole genome. The mutation rate for both SNPs and QTLs were set to $2.5\times {10}^{-8}$ per generation.

#### Sub-setting SNPs and implementation of error rates

The combination of three heritabilities implemented in G0, two scenarios (S1 and S2) and ten independent replicates, has generated 60 independent datasets (Table 1) with their associated pedigree, phenotypic values, and SNP genotypes where genotypes are coded as 0, 1 and 2 for homozygote, heterozygote, and other homozygote, respectively. For each datasets, quality control of genotype was carried out using minor allelic frequency (MAF) of more than 0.01. After quality control, between 46,022 and 46,055 polymorphic SNPs was left in Generation 1 across different replicates and family contributions whilst in Generation 5, the respective count was between 21,725 and 36,999. From the remaining informative SNPs, 20 K marker density was randomly sampled within each scenario and generation. For each of these scenarios, four SNP densities were then considered (0.5 K, 3 K, 10 K and 20 K), generating a total of 240 datasets. Marker panels of 0.5 K, 3 K and 10 K were generated by random sampling from the original SNP panel (20 K).

Finally, for each resulting SNP panel, different genotypic errors were generated with error rates of 0, 1, 5, and 10%, and implemented into genotypic data as follows. To implement errors in genotypic data, a $3 \times 3$ transition probability matrix was assumed:

$${\mathbf{P}} = \left[ \begin{array}{*{20}l} p_{11} &\quad p_{12}  &\quad p_{13} \\ p_{21}  &\quad p_{22}  &\quad p_{23} \\ p_{31} &\quad p_{32} &\quad p_{33}\\ \end{array} \right]$$

where **P** is the transition probability matrix, with elements *p*_*ij*_ being the probability that a biallelic SNP with true genotype *i* (*i* = 1, 2, 3: row) is scored as genotype *j* (*j* = 1, 2, 3: column), where the diagonal elements ($p_{11}$, $p_{22}$ and $p_{33}$) are probabilities of having genotypes (AA, AB, BB) being correctly scored. Simulated scored genotypes were generated from a multinomial distribution using the appropriate row in the matrix **P**. The transition probability matrices used to generate 1, 5 and 10% genotypic errors are shown from left to right in order:

$$\left[\begin{array}{*{20}l}0.990 &\quad 0.006 &\quad 0.004\\ 0.005 &\quad 0.990 &\quad 0.005\\ 0.004 &\quad 0.006 &\quad 0.990\end{array}\right], \left[\begin{array}{*{20}l}0.9500 &\quad 0.003 &\quad 0.0020\\ 0.0025 &\quad 0.950 &\quad 0.0025\\ 0.0020 &\quad 0.003 &\quad 0.9500\end{array}\right], \left[\begin{array}{*{20}l}0.90 &\quad 0.06 &\quad 0.04\\ 0.05 &\quad 0.90 &\quad 0.05\\ 0.04 &\quad 0.06 &\quad 0.90\end{array}\right]$$

In total 960 datasets (2 main scenarios (family contributions) $\times$ 3 heritabilities $\times$ 10 replicates $\times$ 4 SNPs densities $\times$ 4 levels of genotypic errors) were generated for statistical analysis.

### Statistical analysis

#### Estimating the accuracy of prediction

**Figure 1:**
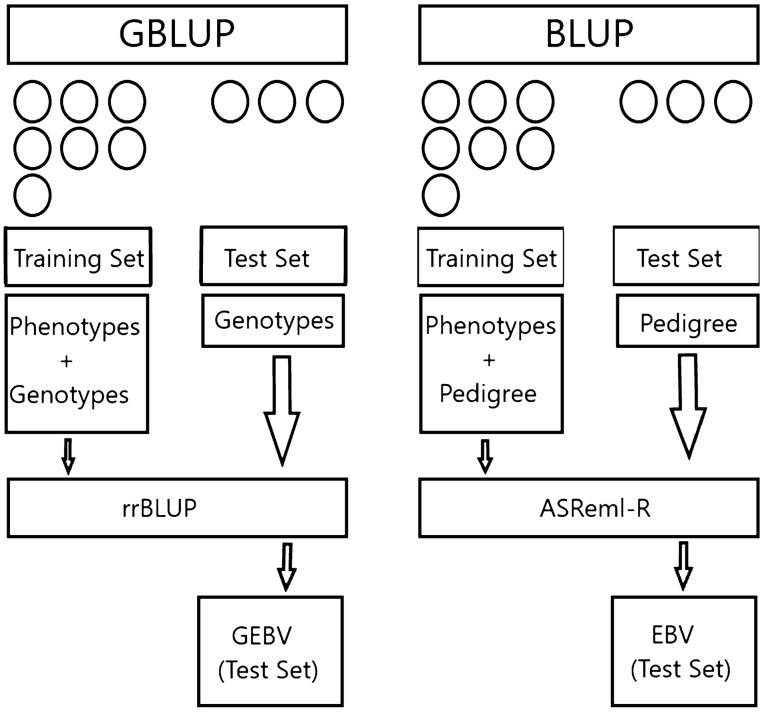
A diagram illustrating how GEBVs and EBVs are calculated where the phenotypic values are unavailable for the selection candidates.

Following the simulation of populations and data preparation, true breeding values (TBV) were calculated by accumulating the QTL and polygenic effects for each individual. Then, the rrBLUP package^52^ and the predict function in ASReml-R^53^ were used to calculate genomic estimated breeding values (GEBVs) and EBVs, respectively. To calculate (G)EBVs, we considered the situation where traits cannot be measured on the selected animals. Consequently, the (G)EBVs of selected candidates were obtained from performance of their sibs. Then, the accuracy of (G)EBVs of candidates were calculated as the Pearson correlation of their (G)EBVs and TBVs. To do that, 30% of progeny per generation were randomly selected as the test set (selection candidates) and the remaining progeny in that generation as the training population (which includes sibs of selection candidates). Next, the phenotypic values of the test population were masked and GEBVs in the test set were obtained using the phenotypic values of their sibs and the GRM of all individuals within each generation.

$${\mathbf{G}} = \frac{{{\mathbf{WW^{\prime}}}}}{c}$$

where ${\mathbf{W}} = \{ W_{ij} \}$ with $W_{ij} = X_{ij} + 1 - 2p_{j}$, ${\mathbf{X}} = \{ X_{ij} \}$ is the matrix of genotypes for individual $i$ and marker $j$, coded as − 1, 0, and 1. $p_{j}$ is considered as the frequency of the first allele at $j^{th}$ marker and $c$ is a constant value equal to $2\sum\nolimits_{j} {p_{j} (1 - p_{j)} }$. Whereas to obtain EBVs in the test set, the phenotypic values of sibs and numerator relationship matrix based on pedigree (NRM) of all individuals in each generation were used. A diagram showing the above-mentioned procedure to obtain (G)EBVs is illustrated in Fig. 1.

#### Descriptive summary analysis of different factors

There were six factors investigated in this simulation study, to assess their effect on accuracy as evaluated using Pearson correlations. These factors and their levels being heritability (*n* = 3); family type (*n* = 2); BV estimation method (*n* = 2); and within the BV = GEBV sets of simulations, genotypic errors (*n* = 4); and marker density (*n* = 4); multiple generations (*n* = 5). In addition, each of these scenarios was evaluated using ten replicates.

Due to the extent of these different factors, the accuracy output was summarised by generating a series of two-way table means, averaging over the replicates and other variables. This was obtained using the aggregate function in the R statistical package^54^. Then, the estimates of prediction and correlation per generation were obtained from the average of all ten replicates of estimates in that generation. Finally, the standard error of accuracies was obtained from standard deviation of accuracies:

$${\text{SE = }}\frac{\sigma }{\sqrt n }$$

where SE is standard error of accuracies, σ is equal to standard deviation of estimates across ten replicates, and *n* is the number of replicates.

#### Estimating the effect of genotypic error on the genomic relationship matrix

The effect of genotypic error on the GRM was investigated using correlation of off-diagonal elements of the GRM without error with off-diagonal elements of matrices obtained from different SNP densities and genotypic error rates. The consideration of off-diagonal elements was due to the fact that they show the genetic relationship between each pairs of individuals. The GRM was constructed according to method of Yang et al.^55^ implemented within the rrBLUP package. Initially the Pearson correlation of off-diagonal elements of GRMs with different error rates were compared. However, the Pearson correlation indicates the extent of linear relatedness, but it does not consider the extent of equality within the pairs. Consequently, calculation of the Lin’s concordance correlation coefficient (CCC)^56^ between the off-diagonal elements of GRMs was also considered as the measurement of reliability. Since Lin’s correlation takes into the account both correlation and correspondence, it is a more reliable estimate for expressing the impact of genotypic errors on GRM. Consequently, the aggregate function was used to summarize the average results over trait heritabilities, replicates and family contributions. All the analysis was carried out using R statistical package^54^.

## Results

### Accuracy of pedigree and genomic-based estimated breeding values

The detailed results of accuracy of genomic predictions, as measured by the correlation between TBVs and EBVs/GEBVs, for different generations, replicates, family type, SNP density and genotypic error rates are provided in Supplementary Table S1. Overall, within each heritability, accuracy of EBVs decreased from Generation 1 to 5. On the other hand, accuracy of GEBVs increased or decreased over the generations, depending on trait heritability, SNP density, genotypic error rate, family type and replicate. For instance, using 0.5 K SNP density and without genotypic error, accuracy of GEBVs decreased across different trait heritabilities over five generations for equal family contribution whilst accuracy increased for unequal family contribution using medium (0.3) and high (0.5) heritability traits. In addition, the accuracy of EBVs increased as heritability increased from 0.05 to 0.5 for equal and unequal family contributions. The same pattern can be noticed for GEBV accuracies. Moreover, for GEBV accuracies, higher SNP density resulted in increase in the accuracy of genomic predictions while increasing the genotypic error rate resulted in a loss of accuracy. The results in the form of two-way summary graphs of mean accuracy across different factors of the study are presented as follows. Nevertheless, the standard errors of (G)EBV accuracies were appreciably small in the Supplementary Table S1 and the figures.

### Accuracy of predictions across family types and generations

**Figure 2:**
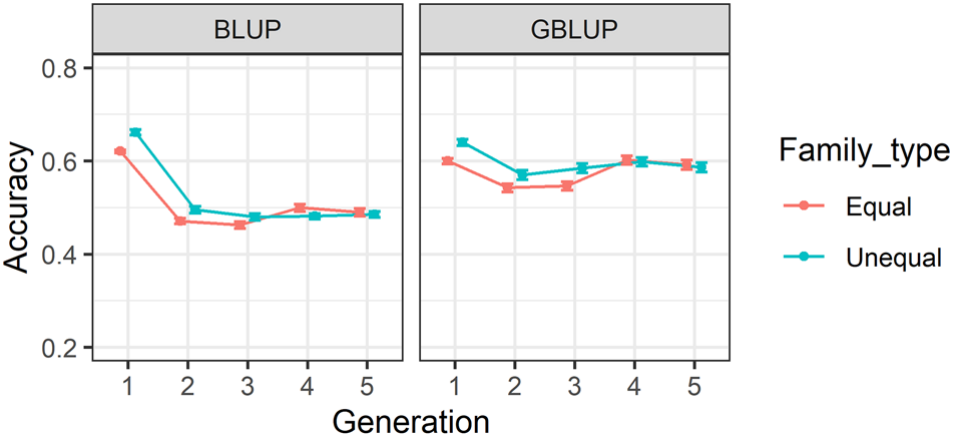
Accuracy of (genomic) estimated breeding values (G)EBV over five generations. The accuracies are provided for two family types (equal and unequal). Accuracies in BLUP were averaged over heritabilities and replicates, while for GBLUP are estimated by averaging correlations over three heritabilities, ten replicates, four marker densities and four genotypic error rates. Standard errors of accuracies are shown as error bars.

Accuracy of (genomic) estimated breeding values using pedigree-BLUP (BLUP) and genomic-BLUP (GBLUP) for equal versus unequal family contribution are provided in Fig. 2, for five consecutive generations. The figure shows that the accuracy of predictions decreased from generation 1 to 2, with a larger decrease for EBV accuracies. However, there was a slight increase in accuracy from Generation 2 to 5 especially for GEBV accuracies. In addition, the accuracy of GEBVs was slightly higher as compared to EBV accuracy, with the exception of Generation 1. Nevertheless, accuracy of predictions provided in Supplementary Table S1 showed that accuracy of (G)EBVs for equal and unequal family contribution scenarios were usually slightly different using high (0.5), medium (0.3) and low (0.05) heritability traits across different SNP densities. However, the difference between the two family types across different generations was small.

### Accuracy of predictions within family types and across heritabilities

**Figure 3:**
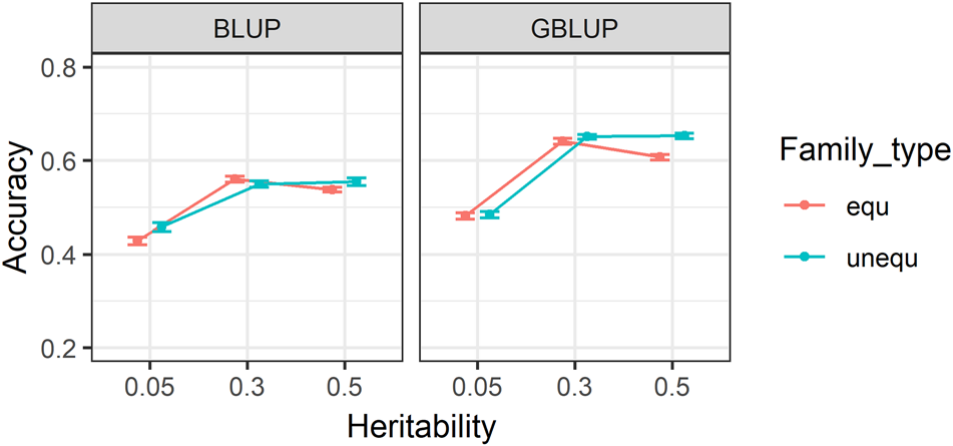
Accuracy of (genomic) estimated breeding values (G)EBV within three heritabilities. The accuracies are provided for equal and unequal family contributions. Accuracies in BLUP were averaged over five generations and ten replicates, while for GBLUP are estimated by averaging correlations over five generations, ten replicates, three marker densities and four genotypic error rates. Standard errors of accuracies are shown as error bars.

Accuracies of EBVs and GEBVs using BLUP and GBLUP, respectively, for equal versus unequal family contribution are given in Fig. 3. The estimates are provided for heritabilities of 0.05, 0.3 and 0.5. The figure shows that accuracy of GEBVs increased with increasing heritability from 0.05 to 0.3. From heritability of 0.3 to 0.5, accuracy of GEBVs for equal family contribution did not change but it decreased for equal family contribution. The accuracy of EBVs increased from heritability of 0.05 to 0.5 for unequal and decreased from heritability of 0.3 to 0.5 for equal family contributions. Nevertheless, family type had a small effect on the accuracies obtained for both EBV and GEBV estimates. However, using individual estimates (Supplementary Table S1), the difference between two family contributions was slightly different and varied across different SNP densities, error rate and replicates. Variation in accuracies of BLUP and GBLUP using equal and unequal family types might be due to variation in size of families across different scenarios with unequal family contribution.

### Accuracy of predictions within heritabilities and across generations

**Figure 4:**
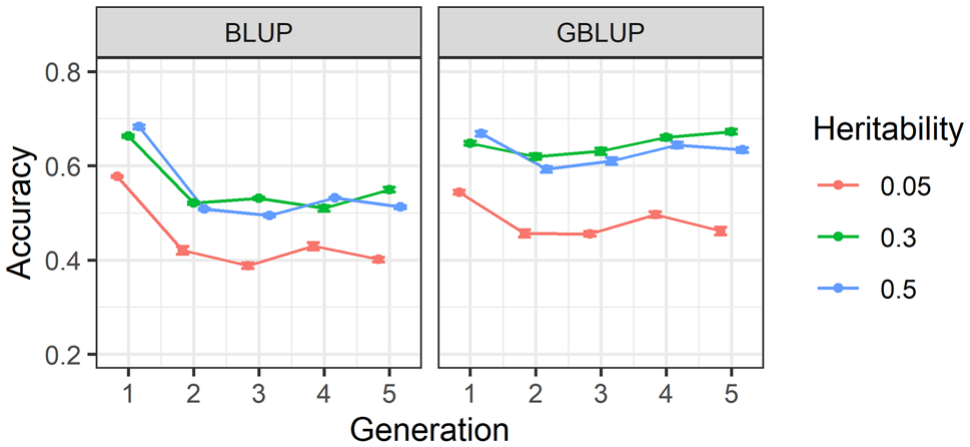
Accuracy of (genomic) estimated breeding values (G)EBV within five generations. The accuracies are provided for heritability of 0.05, 0.3 and 0.5. Accuracies in BLUP were averaged over two family types and ten replicates, while for GBLUP are estimated by averaging correlations over two family types, ten replicates, four marker densities and four genotypic error rates. Standard errors of accuracies are shown as error bars.

Accuracies of EBV and GEBV for heritability of 0.05, 0.3 and 0.5 over five consecutive generation are illustrated in Fig. 4 using BLUP and GBLUP. Accuracy of both EBVs and GEBVs decreased from generation 1 to 2, however, the decline was much higher for EBV accuracies. From generation 2 to 5 accuracy of predictions for both EBVs did not change, whilst for GEBVs using medium (0.3) and high (0.5) heritability accuracy slightly increased. However, there was no change for low (0.05) heritability.

### Effect of genotypic error rates and family types on accuracy of predictions

**Figure 5:**
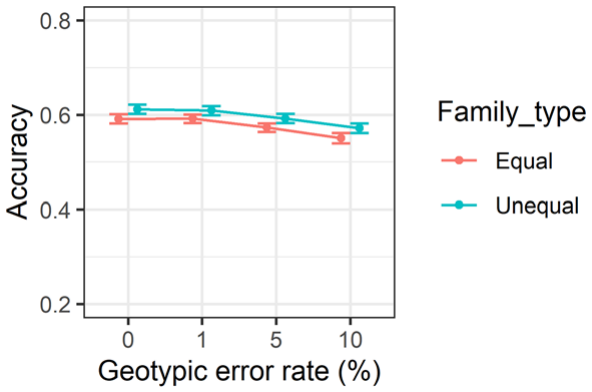
Accuracy of genomic estimated breeding values over genotypic error rates of 0, 1, 5 and 10% for equal versus unequal family contribution. Correlations are averaged over different generations, replicates, SNP densities and heritability. Standard errors of accuracies are shown as error bars.

Variation of GEBV accuracies over different genotypic error rates for equal and unequal family contributions is provided in Fig. 5. Overall, GEBV accuracy has dropped slightly from approximately 0.60 to below 0.58 with increases in genotypic error rate from 1 to 10%. However, family type had little effect on accuracy of predictions, as mentioned before.

### Effect of marker density and family type on accuracy of predictions

**Figure 6:**
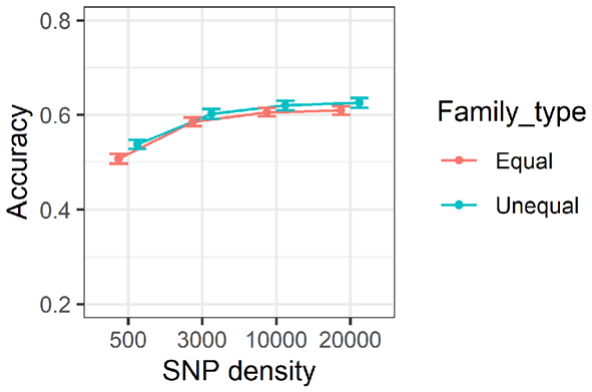
Accuracy of genomic estimated breeding values over marker densities of 0.5, 10 and 20 K for equal versus unequal family contribution. Correlations are averaged over different generations, replicates, genotypic error rates and heritability. Standard errors of accuracies are shown as error bars.

Comparison of accuracy of GEBVs for SNP densities of 0.5 K, 3 K, 10 K and 20 K over equal and unequal family contributions is presented in Fig. 6. The figure illustrates that GEBV accuracy increased as SNP density increased from 0.5 to 20 K. GEBV accuracy increased from above 0.5 to slightly over 0.6, approximately, when the SNP density increased from 0.5 to 20 K. There was a sharp increase in accuracies from 0.5 to 3 K SNP density, followed by a gradual increase in accuracy as SNP density increased from 3 to 10 K and 10 to 20 K. In addition, family type once again showed little effect on the accuracy of genomic predictions. As mentioned, accuracy of genomic prediction provided in Supplementary Table S1 showed that accuracy of GEBVs for the equal family contribution scenario were usually slightly higher using low (0.05), medium (0.3) and high (0.5) heritability traits across different SNP densities, however this was not a general rule across different scenarios and the difference between the two family types across different trait heritabilities was trivial.

### Effect of genotypic errors and heritability on accuracy of predictions

**Figure 7:**
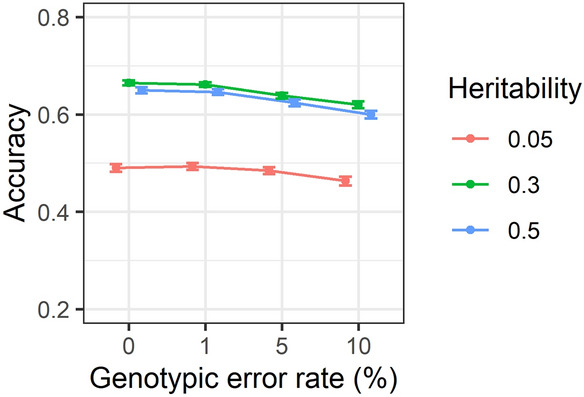
Accuracy of genomic estimated breeding values for genotypic error rates of 0, 1, 5 and 10% over heritabilities of 0.05, 0.3 and 0.5. Correlations are averaged over different generations, replicates, family types and SNP densities. Standard errors of accuracies are shown as error bars.

The variation in accuracy of GEBVs with genotypic error rates of 0, 1, 5 and 10% over the heritabilities of 0.05, 0.3 and 0.5 is depicted in Fig. 7. The results showed that with increasing genotypic error, accuracy of GEBVs decreased gradually within each heritability. However, there was not a big loss in accuracy with increasing error rates. This was especially evident using individual estimates provided in Supplementary Table S1 using more than 3 K SNP density.

### Effect of marker densities and heritability on accuracy of predictions

**Figure 8:**
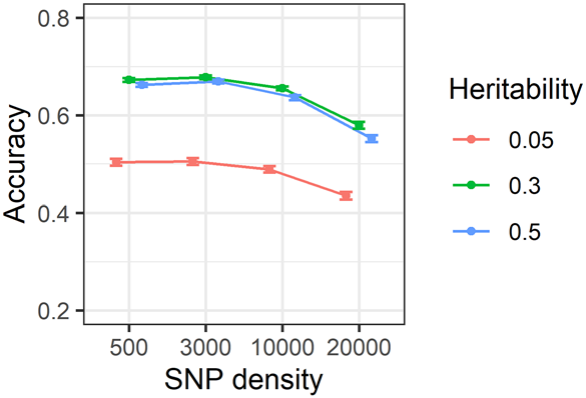
Accuracy of genomic estimated breeding values for 0.5, 3, 10 and 20 K SNP densities over heritabilities of 0.05, 0.3 and 0.5. Correlations are averaged over different generations, replicates, family types and genotypic error rates. Standard errors of accuracies are shown as error bars.

Comparison of GEBV accuracies for SNP densities of 0.5, 3, 10 and 20 K over trait heritabilities of 0.05, 0.3 and 0.5 is depicted in Fig. 8. The results show that increasing the SNP density has resulted in increasing the accuracy of predictions, as does increasing heritability, as reported above.

### Effect of marker densities and genotypic error on accuracy of predictions

**Figure 9:**
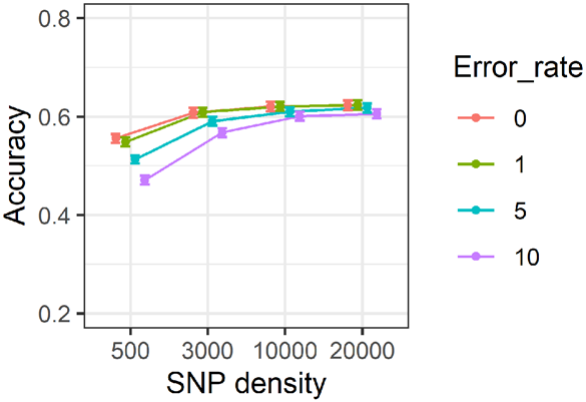
Accuracy of genomic estimated breeding values for 0.5, 3, 10 and 20 K SNP densities over genotypic error rates of 0, 1, 5 and 10%. Correlations are averaged over different generations, replicates, family types and trait heritabilities. Standard errors of accuracies are shown as error bars.

Changes in the accuracy of GEBV for SNP densities of 0.5, 3, 10 and 20 K over genotypic error rates of 0, 1, 5 and 10% are illustrated in Fig. 9. Results showed that accuracies have increased with increasing the SNP density, across different genotypic error rates. In addition, the difference in accuracy between 0 and 10% genotypic error was marginal across 20 K and 10 K SNP densities, whilst the difference was more pronounced for 3 K and 0.5 K SNP density.

### Effect of generation and genotypic error rates on accuracy of predictions

**Figure 10:**
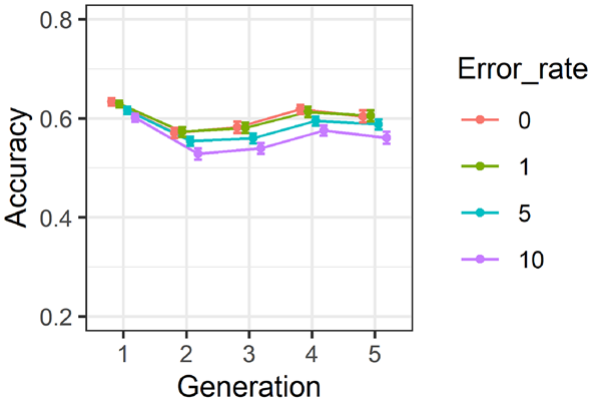
Accuracy of genomic estimated breeding values for five consecutive generation over 0.5, 3, 10 and 20 K SNP densities. Correlations are averaged over different SNP densities, replicates, family types and trait heritabilities. Standard errors of accuracies are shown as error bars.

Changes in the accuracy of GEBV over five consecutive generations for SNP densities of 0.5, 3, 10 and 20 K are illustrated in Fig. 10. Overall, accuracies have decreased from Generation 1 to 2 and then slightly increased from Generation 2 to 5. In addition, the difference between 0 and 1% error was marginal.

### Effect of genotypic error on the genomic relationship matrix

**Figure 11:**
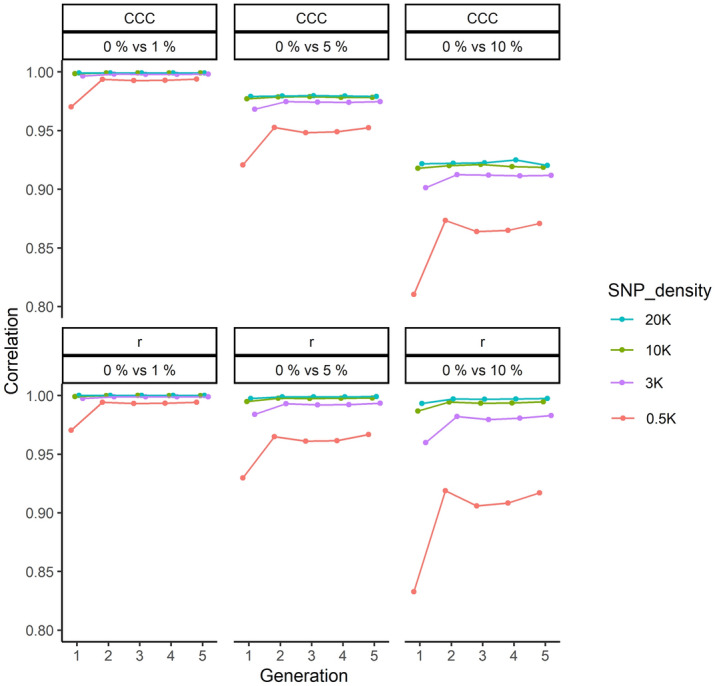
Comparison of off-diagonal elements of genomic relationship matrix (GRM) without error with 1, 5 and 10% error for different SNP densities across five generation. The estimates are averaged across ten replicates, three trait heritabilities (0.5, 0.3 and 0.05) and two family types (equal and unequal). *r* signifies the Pearson correlation and CCC is Lin’s concordance correlation coefficient. In each plot, the correlation between off-diagonal elements of GRM without error and GRM with 1% (0% vs 1%), 5% (0% vs 5%) and 10% error (0% vs 10%) are illustrated. Standard errors were significantly small and were unable to be shown.

A complete list of Pearson and Lin’s correlation coefficients between off-diagonal elements of the GRM without error and those with error rates of 1, 5 and 10% is provided in Supplementary Table S2. The list is organised for both equal and unequal family contributions with three heritabilities (0.5, 0.3 and 0.05) and different marker densities (0.5, 3, 10 and 20 K) in the study. It should be noted that only genotypes within a generation are used for the analysis of GRM not the phenotypic values. Consequently, the results of different scenarios were not affected by heritability or phenotype. However, as each simulation was conducted independently, slight changes in correlation estimates were noticeable for scenarios from one heritability to another. Overall, the outcome of the effect of genotypic error on GRM presented in Supplementary Table S2 suggested that with increasing genotyping error rate, the relatedness between pairs of animals is increasingly under-estimated. To clarify this, the comparison of average correlations is provided as follows: the Pearson and Lin’s correlations of off-diagonal elements of GRM calculated from genotypes with 1, 5 and 10% error and off-diagonal of GRM without error are presented in Fig. 11. The illustration is provided for SNP densities of 0.5, 3, 10 and 20 K across five generations. The correlations are averaged across ten independent replicates, three trait heritabilities (0.5, 0.3 and 0.05) and two family contributions (equal and unequal). The results represented in the figure showed that at 1% genotypic error, both Pearson and Lin’s correlation estimates were similar and high (except in Generation 1). Increasing the genotypic error has resulted in dramatic decrease in estimated correlations as CCC, in particular, small diagonal elements (measures of relatedness) are over-estimated while larger elements are under-estimated (i.e., regression to the mean). In comparison the decrease in Pearson correlation was not as dramatic except for 0.5 K SNP density. In addition, the standard errors were significantly small that could not be shown in the figure.

## Discussion

The current study evaluated the effect of genotypic error on accuracy of genomic prediction and estimation of GRM, and the study was designed to mirror the genome structure of the black tiger prawn reported by Wilson et al.^51^ and population structure of *P. monodon* under captivity as described by Foote et al.^50^. We explored the effects of SNP density, heritability, and family type on accuracy of GEBVs. In addition, the effects of family type and heritability on accuracy of EBVs was also studied, as well as comparisons of accuracies between EBVs and GEBVs. Accuracy of predictions were investigated across five consecutive generations and ten independent replicates in a simulated breeding design. The previously published simulation study on the effect of genotypic error on accuracy of genomic predictions based on genome and population structure of rainbow trout^48^ showed that 10% error had no effect on the accuracy of genomic predictions. However, the current breeding design of black tiger prawns in Australia is different from rainbow trout breeding. Whilst breeding of rainbow trout is based on GS around the globe, in black tiger prawn, it is currently based on mass spawning in the communal rearing environment which would result in presence of unequal contribution of families^57–59^. The existence of unequal family contributions was recently demonstrated using an experiment on the captive breeding of *P. monodon* in Australia^50^. In addition, the genome structure of black tiger prawn is also different from rainbow trout, e.g. the number of chromosomes, making the re-evaluation of study performed by Dufflocq et al.^48^ necessary for black tiger prawn breeding.

### Comparing the accuracy EBVs and GEBVs

The summary analysis over different scenarios in our study showed that the accuracy of GEBVs was on average higher than EBV accuracy when two-way descriptive analysis was performed for family types-generations (Fig. 2), family types-heritabilities (Fig. 3) and heritabilities-generations (Fig. 4) except in generation 1 in which both accuracies were relatively similar. However, detailed accuracy outcomes presented in Supplementary Table S1 showed that the GEBV accuracy can be higher or lower than EBV accuracy, depending on SNP density and genotypic error rate. For example, GBLUP with 10 K and 20 K SNP density, without error has resulted in higher prediction accuracies than BLUP calculations. In contrast, GBLUP with 0.5 K SNP density even without genotypic error has led to lower accuracies compared to BLUP estimations.

Obtaining higher EBV compared to GEBV accuracy using any SNP density or genotypic error rate e.g. from 0.5 K SNP density across different trait heritabilities were in contrast to the results from the simulation study performed on a rainbow trout breeding program^48^. The simulation study on the rainbow trout showed that GBLUP accuracies were normally 8% higher than their corresponding BLUP-based accuracies across heritabilities of 0.1, 0.2 and 0.4 and SNP densities of 0.5 K, 3 K, 7 K and 42 K. Comparing accuracy of prediction using traditional and genomic-based BLUP in several simulations^60–64^ and empirical studies in aquaculture^65–72^ has also demonstrated the higher accuracy values for GEBVs over EBVs. The reason for obtaining higher GEBV compared to EBV accuracies using 0.5 K SNP density can be perhaps due to different experimental design and parameters in other studies as compared to this study. For example, Dufflocq et al.^48^ used full-sib families with a family size of 32 whilst in this study the family size of full-sib families was 100. This allowed more accurate prediction of EBVs in the test population based on the presence of a large number of full-sibs in the training set resulting in more variable EBV estimates compared to GEBVs using 0.5 K SNP density.

### Effect of family type on accuracy of genomic prediction

This study was unable to find any substantial differences between equal and unequal family contributions as presented in Figs. 2, 3, 5 and 6 and Supplementary Table S1. The marginal difference between accuracy of prediction inferred from two different family contributions could be attributed to the availability of a large number of individuals with phenotypic values in the training population. For example, the size of training population for equal family contribution was fixed at 3500 per generation whilst for unequal family contribution it was changing to a medium extent (200–700) in each generation based on the contribution probabilities implemented in the simulation design. As it has been shown, a small to medium increase in the size of the training population, e.g. from 2567 to 2787 in wheat, marginally increased the accuracy of the yield trait from 0.127 to 0.142^73^.

### Effect of heritability on accuracy of genomic prediction

The accuracy of prediction was generally increased at higher trait heritabilities as presented in Fig. 8 and Supplementary Table S1. Specifically, an increase in the heritability from 0.05 to 0.5 has increased the accuracy of GEBV on average by 18% across different scenarios. Higher accuracy of prediction due to increased heritability was as anticipated and has been shown previously^21,48,62^. However, this pattern was not repeated across different SNP densities as presented in Supplementary Table S1. For example, in the first replicate using 0.5 K SNP density without genotypic error in Generation 5, accuracy of GEBVs for heritabilities of 0.05, 0.3 and 0.5 for equal family contributions was recorded as 0.309, 0.584 and 0.578, respectively. This inconsistent pattern could be caused by inconsistency of low-density markers to capture the relationships between individuals as presented in Fig. 11 and Supplementary Table S2. Nevertheless, based on the averages shown in Fig. 8 and Supplementary Table S1, it is very clear in general, even at 0.5 K density, that accuracy increased with increasing heritability.

### Effect of generation on accuracy of genomic prediction

Our results showed that both EBV and GEBV accuracies have decreased over the period of five generations except for GEBV estimates for medium heritability trait (0.3) in which accuracy slightly increased as depicted in Fig. 4. This was in clear contrast to other simulation studies e.g. Nielsen et al.^63^ and Dufflocq et al.^48^. The main difference between our study and the two others; in addition to size of full-sib families, was the mating ratio. Whilst in the current study a mating ratio of 1:1 was implemented, resulting in 50 full-sib families, Dufflocq, et al.^48^ used a 1:3 mating ratio, leading to the production of 120 half-sib families. This has presumably led to a higher chance of better-performing animals to be selected for the next generation. Consequently, this combination of better-performing animals and more families has probably resulted in lower inbreeding, higher additive genetic variation, and better accuracy of prediction across generations^63,64^. Another explanation would be the effect of selection method on accuracies; called the Bulmer effect, and/or the choice of selection method, e.g., selection based on EBVs versus GS^74,75^. The choice of selection can change the extent of LD or unintentionally create low LD, which in turn can change/reduce the accuracy of genomic prediction over generations^63,76^. Otherwise, the difference between our study and the others could be simply due to the extent of relationships between the training and test population. As such, the higher relationship between the two sets can result in higher accuracy of prediction and vice versa^77–79^.

### Effect of SNP density on accuracy of genomic prediction

The outcome of this study in Supplementary Table S1 has shown that the accuracy of GEBVs has increased with increasing the SNP densities in individual comparisons. The elevation of accuracy due to increasing the SNP density was in agreement with the outcome of a simulation study of rainbow trout^48^ and results of empirical studies on accuracy of prediction for skin and fillet colour^80^ and, disease resistance^65,68^ in Atlantic salmon. In addition, our results showed that accuracy of GEBVs did not increase significantly beyond 10 K SNP density. The outcome of this study was also in agreement with results of an empirical study on accuracy of disease resistance in rainbow trout^71^ and Atlantic salmon^67^ which showed that SNP densities of more than 10 K did not have a meaningful effect on increasing the accuracy of genomic predictions. However, the effect of higher marker densities, e.g., 50 or 100 K, on accuracy of genomic predictions was not evaluated against 10 or 20 K SNP densities. In addition, in some scenarios especially for medium (0.3) and high (0.5) trait heritabilities, even 3 K SNP density efficiently estimated the accuracies. Consequently, it can only be concluded that there was a marginal difference between accuracy of prediction using 3 K and 20 K SNP densities within this study especially when the genotypic errors was relatively low (< 5%).

### Effect of genotypic error on accuracy of GEBV and GRM

The descriptive summary analysis results showed that when the genotypic error increased from 0 to 10%, accuracy of GEBVs decreased by approximately 6% and 7% across different heritabilities (Fig. 7) and generations (Fig. 10), respectively. Overall, the presence of 10% error only had a marginal impact on accuracy of predictions using more than 10 K SNP density as presented in Fig. 9. In addition, based on the results of individual estimations provided in Supplementary Table S1, increasing the error rate from 0 to 10% has resulted in decreasing the GEBV accuracy on average by 20% across different scenarios. The presence of 10% error did not have a substantial impact on accuracy of GEBVs using higher than 10 K SNP density, however, its effect on accuracy using a lower density SNP panel, particularly 0.5 K SNP density, was more pronounced. The results of both the two-way summary analysis and individual estimates were in clear contrast to those reported by Dufflocq et al.^48^ where no substantial difference between accuracy of genomic predictions in the presence of 0% and 10% genotypic errors across different marker densities was reported.

Overall, the Pearson correlations displayed in Fig. 11 for scenarios with 10% error in genotypic data indicate that this level of error may not under-estimate the relatedness. However, individual correlations presented in Supplementary Table S2 suggested that the presence of 10% error with 10 K or higher marker density would result in better accuracy of GEBVs compared to BLUP accuracies using medium (0.3) and high (0.5) trait heritabilities. Whilst Lin’s correlations would imply that 10% error could dramatically underestimate or overestimate the relationship between individuals as depicted in Fig. 11, individual Lin’s correlations presented in Supplementary Table S2 showed that presence of as much as 10% error using higher than 10 K SNP density had marginal effect on relatedness between individuals. However, occurrence of 10% error in the 3 and 0.5 K SNP panel could have negative effects on accuracy of genomic prediction in breeding designs, at least for the combination of population and genome structure provided in this study.

### Implication for design of breeding programs

Currently, a lot of attention in breeding design is being directed to imputation methodology to reduce the cost of genotyping. This study has suggested that the presence of up to 5% genotypic error even with application of 0.5 K SNP panel might not be problematic. Moreover, the presence of up to 10% errors in a 10 and 20 K panel might not have severe impact on accuracy of predictions.

There are several solutions to reduce the effect of genotypic error on accuracy of genomic prediction. A small proportion of sporadic errors in genotypic data can be rectified using different imputation methods. However, this could be only possible if the existence of error is known/detected. Application of higher marker density could be another option. This can be a viable alternative if genotypic data do not have higher error rates. However, using higher marker density genotypes can also increase the cost genotyping. Another alternative could be the implementation of a genotypic error term in algorithms and statistical analysis to deal with random misclassification errors^33,40,41^, however this approach would require repeated genotyping^34^.

Overall, the results of this study suggested that the presence of genotypic errors, as low as 5%, can negatively impact the relationships in the GRM and accuracy of genomic predictions if lower than 10 K SNP density is used. Below an error rate of 5% (1% specifically), there was little effect of reducing the accuracy, and the correlation between off-diagonal of GRMs either using Pearson or Lin’s correlations remained high, even using 0.5 K SNP density. As mentioned, random errors can be captured using LD or HWE analysis, pedigree reconstruction, comparison with high-quality reference genotypes, etc., as well as additional analysis such as quality control checking for Mendelian inheritance^81,82^ or incorporating weighted analysis for read depth^83,84^. However, even if the presence of errors is detected, the correction of such errors would be time consuming, expensive and a complicated practice. Consequently, where feasible, the better alternative could be to avoid generating errors, e.g., by collection of high-quality samples, reduction of the laboratory-related errors, e.g., environmental contamination, and using precise sequencing procedures.

## Supplementary Information

Supplementary Information.
