# Author Correction: Impact of genotypic errors with equal and unequal family contribution on accuracy of genomic prediction in aquaculture using simulation

**DOI:** 10.1038/s41598-021-99302-z

**Published:** 2021-09-29

**Authors:** N. Khalilisamani, P. C. Thomson, H. W. Raadsma, M. S. Khatka

**Affiliations:** 1 ARC Research Hub for Advanced Prawn Breeding, James Cook University, Townsville, QLD 4811 Australia; 2 Sydney School of Veterinary Science, Faculty of Science, The University of Sydney, Camden, NSW 2570 Australia; 3 School of Life and Environmental Sciences, Faculty of Science, The University of Sydney, Camden, NSW 2570 Australia

Correction to: *Scientific Reports* https://doi.org/10.1038/s41598-021-97873-5, published online 15 September 2021

The original version of this Article contained an error in the horizontal axis label of Figure 3. As a result,

**Figure 3:**
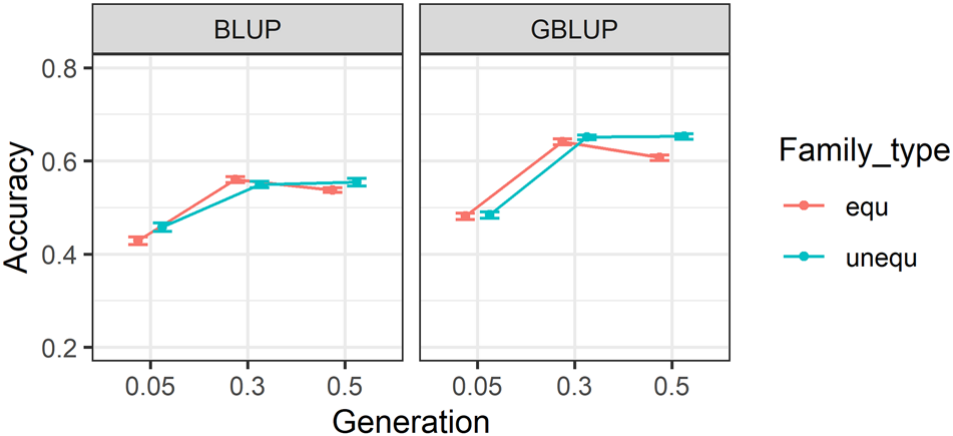
Accuracy of (genomic) estimated breeding values (G)EBV within three heritabilities. The accuracies are provided for equal and unequal family contributions. Accuracies in BLUP were averaged over five generations and ten replicates, while for GBLUP are estimated by averaging correlations over five generations, ten replicates, three marker densities and four genotypic error rates. Standard errors of accuracies are shown as error bars.

"Generation"

now reads:

"Heritability"

The original Figure 3 and accompanying legend appear below.

The original Article has been corrected.

