# Supplementary material for: Impact of genotypic errors with equal and unequal family contribution on accuracy of genomic prediction in aquaculture using simulation
Source: Sci Rep. 2021 Sep 15;11:18318. doi: 10.1038/s41598-021-97873-5 (PMC8443606; doi:10.1038/s41598-021-97873-5)
Supplement: Supplementary file 1 — Supplementary Information. [file 41598_2021_97873_MOESM1_ESM.pdf]

Impact of genotypic errors with equal and unequal family contribution on accuracy of genomic prediction in aquaculture using simulation

N. Khalilisamani[1,2], P. C. Thomson[1,3], H. W. Raadsma[1,2] and M. S. Khatkar[1,2]

[1]ARC Research Hub for Advanced Prawn Breeding, James Cook University, Townsville QLD 4811 Australia

[2] The University of Sydney, Sydney School of Veterinary Science, Faculty of Science, Camden NSW 2570 Australia

[3] The University of Sydney, School of Life and Environmental Sciences, Faculty of Science, Camden NSW 2570 Australia.

Supplementary Table S1. Accuracy of (genomic) estimated breeding values for equal and equal family contribution. The results are presented for five generations, averaged over ten independent replicates using SNP densities of 500 (0.5 K), 3000 (3 K), 10000 (10 K) and 20000 (20 K) and, genotypic error rates of 0 (without error), 1, 5 and 10 %. The numbers in parenthesis are standard errors using ten replicates.

Generation	SNP Density	Error (%)	BLUP Type	Equal Family Contribution			Unequal Family Contribution		
				h^2: 0.05	h^2: 0.3	h^2: 0.5	h^2: 0.05	h^2: 0.3	h^2: 0.5
1	-	-	BLUP	0.577 (0.01)	0.646 (0.01)	0.640 (0.00)	0.577 (0.01)	0.681 (0.01)	0.726 (0.00)
2	-	-	BLUP	0.402 (0.02)	0.504 (0.01)	0.508 (0.01)	0.440 (0.04)	0.538 (0.01)	0.509 (0.01)
3	-	-	BLUP	0.374 (0.01)	0.538 (0.01)	0.476 (0.00)	0.402 (0.03)	0.524 (0.00)	0.514 (0.01)
4	-	-	BLUP	0.405 (0.02)	0.555 (0.01)	0.539 (0.00)	0.454 (0.03)	0.464 (0.02)	0.526 (0.00)
5	-	-	BLUP	0.386 (0.01)	0.559 (0.02)	0.526 (0.01)	0.417 (0.02)	0.540 (0.01)	0.499 (0.01)
1	500	0	GBLUP	0.496 (0.01)	0.606 (0.00)	0.593 (0.00)	0.537 (0.01)	0.623 (0.01)	0.681 (0.01)
2	500	0	GBLUP	0.399 (0.02)	0.562 (0.01)	0.536 (0.02)	0.454(0.03)	0.613 (0.01)	0.565 (0.00)
3	500	0	GBLUP	0.406 (0.02)	0.583 (0.03)	0.518 (0.01)	0.450 (0.02)	0.622 (0.02)	0.609 (0.01)
4	500	0	GBLUP	0.476 (0.02)	0.662 (0.01)	0.572 (0.01)	0.477 (0.02)	0.615 (0.01)	0.632 (0.01)
5	500	0	GBLUP	0.442 (0.04)	0.653 (0.01)	0.589 (0.00)	0.433 (0.02)	0.653 (0.00)	0.615 (0.01)
1	3000	0	GBLUP	0.554 (0.01)	0.657 (0.00)	0.648 (0.00)	0.567 (0.01)	0.671 (0.01)	0.718 (0.01)
2	3000	0	GBLUP	0.449 (0.02)	0.634 (0.01)	0.599 (0.02)	0.477 (0.03)	0.659 (0.01)	0.634 (0.01)
3	3000	0	GBLUP	0.453 (0.01)	0.655 (0.02)	0.613 (0.01)	0.479 (0.02)	0.663 (0.01)	0.676 (0.01)
4	3000	0	GBLUP	0.521 (0.02)	0.705 (0.01)	0.657 (0.01)	0.509 (0.02)	0.660 (0.01)	0.696 (0.01)
5	3000	0	GBLUP	0.480 (0.04)	0.702 (0.01)	0.656 (0.00)	0.459 (0.02)	0.703 (0.00)	0.676 (0.01)
1	10000	0	GBLUP	0.573 (0.01)	0.669 (0.00)	0.663 (0.00)	0.578 (0.01)	0.690 (0.01)	0.737 (0.01)
2	10000	0	GBLUP	0.465 (0.02)	0.641 (0.01)	0.624 (0.02)	0.485 (0.03)	0.675 (0.01)	0.655 (0.00)

3	10000	0	GBLUP	0.463 (0.01)	0.666 (0.02)	0.630 (0.00)	0.484 (0.02)	0.682 (0.01)	0.694 (0.01)
4	10000	0	GBLUP	0.536 (0.01)	0.716 (0.01)	0.675 (0.01)	0.509 (0.02)	0.681 (0.01)	0.705 (0.01)
5	10000	0	GBLUP	0.485 (0.03)	0.708 (0.01)	0.667 (0.00)	0.461 (0.02)	0.705 (0.00)	0.684 (0.01)
1	20000	0	GBLUP	0.577 (0.01)	0.673 (0.00)	0.667 (0.00)	0.580 (0.01)	0.693 (0.01)	0.746 (0.01)
2	20000	0	GBLUP	0.467 (0.02)	0.645 (0.01)	0.632 (0.02)	0.488 (0.03)	0.679 (0.01)	0.659 (0.00)
3	20000	0	GBLUP	0.465 (0.01)	0.666 (0.02)	0.633 (0.00)	0.479 (0.02)	0.683 (0.01)	0.697 (0.01)
4	20000	0	GBLUP	0.538 (0.01)	0.718 (0.01)	0.678 (0.01)	0.507 (0.02)	0.684 (0.01)	0.707 (0.01)
5	20000	0	GBLUP	0.489 (0.03)	0.709 (0.01)	0.669 (0.00)	0.461 (0.02)	0.706 (0.00)	0.685 (0.01)
1	500	1	GBLUP	0.484 (0.01)	0.596 (0.00)	0.591 (0.01)	0.524 (0.01)	0.621 (0.01)	0.677 (0.00)
2	500	1	GBLUP	0.417 (0.02)	0.549 (0.01)	0.523 (0.02)	0.448 (0.03)	0.603 (0.01)	0.556 (0.00)
3	500	1	GBLUP	0.424 (0.01)	0.572 (0.03)	0.501 (0.01)	0.448 (0.02)	0.609 (0.02)	0.595 (0.01)
4	500	1	GBLUP	0.446 (0.02)	0.651 (0.01)	0.560 (0.01)	0.469 (0.02)	0.604 (0.01)	0.621 (0.01)
5	500	1	GBLUP	0.469 (0.03)	0.646 (0.02)	0.574 (0.00)	0.430 (0.02)	0.639 (0.00)	0.601 (0.01)
1	3000	1	GBLUP	0.537 (0.01)	0.652 (0.00)	0.644 (0.00)	0.565 (0.01)	0.675 (0.01)	0.715 (0.01)
2	3000	1	GBLUP	0.475 (0.02)	0.629 (0.01)	0.616 (0.01)	0.475 (0.03)	0.670 (0.01)	0.630 (0.01)
3	3000	1	GBLUP	0.467 (0.01)	0.652 (0.02)	0.614 (0.00)	0.477 (0.02)	0.668 (0.01)	0.670 (0.01)
4	3000	1	GBLUP	0.512 (0.02)	0.703 (0.01)	0.666 (0.01)	0.509 (0.02)	0.662 (0.01)	0.690 (0.01)
5	3000	1	GBLUP	0.507 (0.03)	0.698 (0.01)	0.654 (0.00)	0.460 (0.02)	0.695 (0.00)	0.672 (0.01)
1	10000	1	GBLUP	0.561 (0.01)	0.668 (0.00)	0.663 (0.00)	0.578 (0.01)	0.689 (0.01)	0.735 (0.01)
2	10000	1	GBLUP	0.490 (0.01)	0.640 (0.01)	0.620 (0.02)	0.486 (0.03)	0.675 (0.01)	0.651 (0.00)
3	10000	1	GBLUP	0.472 (0.01)	0.663 (0.02)	0.626 (0.00)	0.484 (0.02)	0.680 (0.01)	0.691 (0.01)
4	10000	1	GBLUP	0.529 (0.02)	0.713 (0.01)	0.673 (0.01)	0.508 (0.02)	0.678 (0.01)	0.702 (0.01)
5	10000	1	GBLUP	0.512 (0.03)	0.706 (0.01)	0.665 (0.00)	0.460 (0.02)	0.702 (0.00)	0.682 (0.01)
1	20000	1	GBLUP	0.567 (0.01)	0.672 (0.00)	0.665 (0.00)	0.581 (0.01)	0.692 (0.01)	0.744 (0.01)
2	20000	1	GBLUP	0.492 (0.01)	0.645 (0.01)	0.629 (0.02)	0.487 (0.03)	0.680 (0.01)	0.657 (0.00)
3	20000	1	GBLUP	0.476 (0.01)	0.667 (0.02)	0.632 (0.00)	0.480 (0.02)	0.682 (0.01)	0.695 (0.01)
4	20000	1	GBLUP	0.530 (0.02)	0.718 (0.01)	0.677 (0.01)	0.507 (0.02)	0.682 (0.01)	0.706 (0.01)
5	20000	1	GBLUP	0.515 (0.03)	0.709 (0.01)	0.669 (0.00)	0.461 (0.02)	0.705 (0.00)	0.684 (0.01)
1	500	5	GBLUP	0.447 (0.01)	0.578 (0.01)	0.571 (0.00)	0.511 (0.01)	0.598 (0.01)	0.644 (0.01)
2	500	5	GBLUP	0.402 (0.01)	0.500 (0.01)	0.486 (0.02)	0.419 (0.03)	0.557 (0.01)	0.516 (0.00)
3	500	5	GBLUP	0.397 (0.02)	0.498 (0.02)	0.458 (0.01)	0.414 (0.02)	0.563 (0.01)	0.546 (0.01)
4	500	5	GBLUP	0.435 (0.02)	0.590 (0.02)	0.519 (0.01)	0.452 (0.02)	0.559 (0.01)	0.576 (0.01)

5	500	5	GBLUP	0.464 (0.03)	0.583 (0.02)	0.540 (0.00)	0.419 (0.02)	0.593 (0.00)	0.550 (0.01)
1	3000	5	GBLUP	0.523 (0.01)	0.641 (0.00)	0.630 (0.00)	0.558 (0.01)	0.661 (0.01)	0.701 (0.01)
2	3000	5	GBLUP	0.470 (0.02)	0.604 (0.01)	0.568 (0.02)	0.463 (0.03)	0.650 (0.01)	0.605 (0.00)
3	3000	5	GBLUP	0.463 (0.01)	0.629 (0.03)	0.569 (0.01)	0.465 (0.02)	0.654 (0.01)	0.643 (0.01)
4	3000	5	GBLUP	0.509 (0.02)	0.686 (0.01)	0.627 (0.01)	0.499 (0.02)	0.648 (0.01)	0.665 (0.01)
5	3000	5	GBLUP	0.504 (0.03)	0.679 (0.01)	0.626 (0.00)	0.449 (0.02)	0.677 (0.01)	0.642 (0.01)
1	10000	5	GBLUP	0.559 (0.01)	0.653 (0.01)	0.657 (0.00)	0.573 (0.01)	0.671 (0.01)	0.727 (0.01)
2	10000	5	GBLUP	0.487 (0.01)	0.632 (0.01)	0.608 (0.02)	0.482 (0.03)	0.656 (0.01)	0.633 (0.00)
3	10000	5	GBLUP	0.471 (0.01)	0.634 (0.02)	0.613 (0.00)	0.481 (0.02)	0.660 (0.01)	0.678 (0.01)
4	10000	5	GBLUP	0.523 (0.02)	0.689 (0.01)	0.666 (0.01)	0.504 (0.02)	0.661 (0.01)	0.690 (0.01)
5	10000	5	GBLUP	0.509 (0.03)	0.685 (0.01)	0.654 (0.00)	0.456 (0.02)	0.703 (0.00)	0.670 (0.01)
1	20000	5	GBLUP	0.567 (0.01)	0.659 (0.01)	0.657 (0.00)	0.578 (0.01)	0.686 (0.01)	0.739 (0.01)
2	20000	5	GBLUP	0.491 (0.01)	0.638 (0.01)	0.620 (0.01)	0.487 (0.03)	0.675 (0.01)	0.650 (0.00)
3	20000	5	GBLUP	0.476 (0.01)	0.639 (0.02)	0.628 (0.00)	0.482 (0.02)	0.679 (0.01)	0.689 (0.01)
4	20000	5	GBLUP	0.531 (0.02)	0.695 (0.01)	0.665 (0.01)	0.507 (0.02)	0.680 (0.01)	0.698 (0.01)
5	20000	5	GBLUP	0.515 (0.03)	0.693 (0.01)	0.661 (0.00)	0.458 (0.02)	0.703 (0.00)	0.678 (0.01)
1	500	10	GBLUP	0.417 (0.01)	0.550 (0.00)	0.528 (0.00)	0.464 (0.01)	0.567 (0.01)	0.629 (0.01)
2	500	10	GBLUP	0.338 (0.02)	0.441 (0.01)	0.443 (0.01)	0.382 (0.03)	0.511 (0.01)	0.458 (0.00)
3	500	10	GBLUP	0.366 (0.03)	0.475 (0.03)	0.425 (0.01)	0.380 (0.01)	0.520 (0.02)	0.492 (0.02)
4	500	10	GBLUP	0.391 (0.03)	0.574 (0.01)	0.478 (0.01)	0.431 (0.01)	0.496 (0.01)	0.534 (0.01)
5	500	10	GBLUP	0.356 (0.04)	0.568 (0.02)	0.485 (0.00)	0.366 (0.03)	0.555 (0.01)	0.500 (0.00)
1	3000	10	GBLUP	0.531 (0.01)	0.622 (0.00)	0.609 (0.01)	0.538 (0.00)	0.635 (0.01)	0.684 (0.01)
2	3000	10	GBLUP	0.417 (0.03)	0.586 (0.01)	0.550 (0.01)	0.4498 (0.03)	0.625 (0.01)	0.580 (0.01)
3	3000	10	GBLUP	0.431 (0.01)	0.603 (0.02)	0.550 (0.00)	0.464 (0.02)	0.621 (0.01)	0.600 (0.01)
4	3000	10	GBLUP	0.498 (0.02)	0.658 (0.01)	0.603 (0.01)	0.489 (0.02)	0.619 (0.01)	0.628 (0.01)
5	3000	10	GBLUP	0.458 (0.04)	0.655 (0.01)	0.600 (0.00)	0.446 (0.02)	0.649 (0.01)	0.621 (0.01)
1	10000	10	GBLUP	0.568 (0.01)	0.655 (0.01)	0.637 (0.00)	0.572 (0.01)	0.670 (0.01)	0.714 (0.01)
2	10000	10	GBLUP	0.454 (0.02)	0.613 (0.01)	0.610 (0.01)	0.471 (0.03)	0.651 (0.01)	0.615 (0.00)
3	10000	10	GBLUP	0.454 (0.01)	0.639 (0.02)	0.592 (0.00)	0.482 (0.02)	0.657 (0.01)	0.662 (0.01)
4	10000	10	GBLUP	0.530 (0.02)	0.698 (0.01)	0.648 (0.01)	0.500 (0.02)	0.658 (0.01)	0.670 (0.01)
5	10000	10	GBLUP	0.477 (0.03)	0.690 (0.01)	0.642 (0.00)	0.458 (0.02)	0.677 (0.00)	0.654 (0.01)
1	20000	10	GBLUP	0.565 (0.01)	0.653 (0.00)	0.643 (0.01)	0.571 (0.01)	0.682 (0.01)	0.728 (0.01)

2	20000	10	GBLUP	0.464 (0.02)	0.629 (0.01)	0.602 (0.01)	0.480 (0.03)	0.666 (0.01)	0.638 (0.00)
3	20000	10	GBLUP	0.468 (0.01)	0.631 (0.02)	0.611 (0.00)	0.475 (0.02)	0.671 (0.01)	0.675 (0.01)
4	20000	10	GBLUP	0.511 (0.01)	0.687 (0.01)	0.652 (0.01)	0.501 (0.02)	0.667 (0.01)	0.687 (0.01)
5	20000	10	GBLUP	0.460 (0.03)	0.678 (0.01)	0.649 (0.00)	0.456 (0.02)	0.693 (0.00)	0.667 (0.01)

Supplementary Table S2. Correlation of off-diagonal elements of genomic relationship matrix without genotypic error with off-diagonal of relationship matrices with 1, 5 and 10 % error. The correlations are provided for equal and equal family contribution, trait heritabilities of 0.5, 0.3 and 0.05 and, marker densities of 0.5 K (500), 3 K (3000), 10 (10000) and 20 K (20000). The results have averaged over ten independent replicates. The numbers in parenthesis are the standard errors over ten replicates.

**Generation**	**Heritability**	**SNP Density**	**Correlation Type**	**Equal Family Contribution**			**Unequal Family Contribution**		
				**1 %**	**5 %**	**10 %**	**1 %**	**5 %**	**10 %**
1	0.5	20000	***r ****	1.00 (0.00)	0.997 (0.00)	0.992 (0.00)	1.00 (0.00)	0.998 (0.00)	0.994 (0.00)
2	0.5	20000	***r ****	1.00 (0.00)	0.999 (0.00)	0.998 (0/00)	1.00 (0.00)	0.999 (0.00)	0.997 (0.00)
3	0.5	20000	***r ****	1.00 (0.00)	0.999 (0.00)	0.997 (0.00)	1.00 (0.00)	0.999 (0.00)	0.997 (0.00)
4	0.5	20000	***r ****	1.00 (0.00)	0.999 (0.00)	0.996 (0.00)	1.00 (0.00)	0.999 (0.00)	0.997 (0.00)
5	0.5	20000	***r ****	1.00 (0.00)	0.999 (0.00)	0.997 (0.00)	1.00 (0.00)	0.999 (0.00)	0.997 (0.00)
1	0.5	10000	***r ****	0.999 (0.00)	0.994 (0.00)	0.985 (0.00)	0.999 (0.00)	0.996 (0.00)	0.989 (0.00)
2	0.5	10000	***r ****	1.00 (0.00)	0.998 (0.00)	0.995 (0.00)	1.00 (0.00)	0.998 (0.00)	0.995 (0.00)
3	0.5	10000	***r ****	1.00 (0.00)	0.998 (0.00)	0.994 (0.00)	1.00 (0.00)	0.998 (0.00)	0.994 (0.00)
4	0.5	10000	***r ****	1.00 (0.00)	0.997 (0.00)	0.993 (0.00)	1.00 (0.00)	0.998 (0.00)	0.993 (0.00)
5	0.5	10000	***r ****	1.00 (0.00)	0.998 (0.00)	0.994 (0.00)	1.00 (0.00)	0.997 (0.00)	0.993 (0.00)
1	0.5	3000	***r ****	0.997 (0.00)	0.982 (0.00)	0.955 (0.00)	0.998 (0.00)	0.986 (0.00)	0.965 (0.00)
2	0.5	3000	***r ****	0.999 (0.00)	0.994 (0.00)	0.984 (0.00)	0.999 (0.00)	0.993 (0.00)	0.983 (0.00)
3	0.5	3000	***r ****	0.999 (0.00)	0.993 (0.00)	0.982 (0.00)	0.999 (0.00)	0.993 (0.00)	0.981 (0.00)
4	0.5	3000	***r ****	0.999 (0.00)	0.992 (0.00)	0.980 (0.00)	0.999 (0.00)	0.992 (0.00)	0.980 (0.00)
5	0.5	3000	***r ****	0.999 (0.00)	0.993 (0.00)	0.982 (0.00)	0.999 (0.99)	0.992 (0.00)	0.978 (0.00)

1	0.5	500	***r****	0.987 (0.00)	0.928 (0.00)	0.839 (0.00)	0.965 (0.02)	0.947 (0.01)	0.841 (0.02)
2	0.5	500	***r****	0.995 (0.00)	0.971 (0.00)	0.928 (0.00)	0.995 (0.00)	0.966 (0.00)	0.925 (0.00)
3	0.5	500	***r****	0.994 (0.00)	0.965 (0.00)	0.914 (0.01)	0.994 (0.00)	0.962 (0.00)	0.911 (0.01)
4	0.5	500	***r****	0.993 (0.00)	0.960 (0.00)	0.901 (0.00)	0.994 (0.00)	0.960 (0.00)	0.905 (0.01)
5	0.5	500	***r****	0.994 (0.00)	0.965 (0.00)	0.914 (0.00)	0.993 (0.00)	0.959 (0.00)	0.896 (0.00)
1	0.3	20000	***r****	1.00 (0.00)	0.997 (0.00)	0.993 (0.00)	1.00 (0.00)	0.998 (0.00)	0.994 (0.00)
2	0.3	20000	***r****	1.00 (0.00)	0.999 (0.00)	0.997 (0.00)	1.00 (0.00)	1.00 (0.00)	0.996 (0.00)
3	0.3	20000	***r****	1.00 (0.00)	0.999 (0.00)	0.997 (0.00)	1.00 (0.00)	0.999 (0.00)	0.997 (0.00)
4	0.3	20000	***r****	1.00 (0.00)	0.998 (0.00)	0.996 (0.00)	1.00 (0.00)	0.999 (0.00)	0.999 (0.00)
5	0.3	20000	***r****	1.00 (0.00)	0.999 (0.00)	0.997 (0.00)	1.00 (0.00)	0.999 (0.00)	0.998 (0.00)
1	0.3	10000	***r****	0.999 (0.00)	0.994 (0.00)	0.985 (0.00)	0.999 (0.00)	0.995 (0.00)	0.989 (0.00)
2	0.3	10000	***r****	1.00 (0.00)	0.998 (0.00)	0.994 (0.00)	1.00 (0.00)	0.997 (0.00)	0.995 (0.00)
3	0.3	10000	***r****	1.00 (0.00)	0.998 (0.00)	0.994 (0.00)	1.00 (0.00)	0.998 (0.00)	0.994 (0.00)
4	0.3	10000	***r****	0.999 (0.00)	0.997 (0.00)	0.992 (0.00)	1.00 (0.00)	0.998 (0.00)	0.994 (0.00)
5	0.3	10000	***r****	1.00 (0.00)	0.998 (0.00)	0.994 (0.00)	1.00 (0.00)	0.998 (0.00)	0.995 (0.00)
1	0.3	3000	***r****	0.997 (0.00)	0.982 (0.00)	0.956 (0.00)	0.998 (0.00)	0.986 (0.00)	0.964 (0.00)
2	0.3	3000	***r****	0.999 (0.00)	0.993 (0.00)	0.981 (0.00)	0.999 (0.00)	0.992 (0.00)	0.979 (0.00)
3	0.3	3000	***r****	0.999 (0.00)	0.993 (0.00)	0.982 (0.00)	0.999 (0.00)	0.992 (0.00)	0.981 (0.00)
4	0.3	3000	***r****	0.998 (0.00)	0.989 (0.00)	0.974 (0.00)	0.999 (0.00)	0.9934(0.00)	0.984 (0.00)
5	0.3	3000	***r****	0.999 (0.00)	0.992 (0.00)	0.980 (0.00)	0.999 (0.00)	0.994 (0.00)	0.985 (0.00)
1	0.3	500	***r****	0.987 (0.00)	0.927 (0.00)	0.838 (0.00)	0.963 (0.03)	0.939 (0.00)	0.835 (0.02)
2	0.3	500	***r****	0.994 (0.00)	0.965 (0.00)	0.914 (0.01)	0.993 (0.00)	0.958 (0.01)	0.906 (0.01)
3	0.3	500	***r****	0.994 (0.00)	0.963 (0.00)	0.913 (0.00)	0.994 (0.00)	0.963 (0.00)	0.910 (0.00)
4	0.3	500	***r****	0.991 (0.00)	0.950 (0.00)	0.886 (0.01)	0.994 (0.00)	0.969 (0.00)	0.920 (0.01)
5	0.3	500	***r****	0.993 (0.00)	0.960 (0.00)	0.906 (0.00)	0.995 (0.00)	0.969 (0.00)	0.921 (0.00)
1	0.05	20000	***r****	1.00 (0.00)	0.997 (0.00)	0.993 (0.00)	1.00 (0.00)	0.998 (0.00)	0.994 (0.00)
2	0.05	20000	***r****	1.00 (0.00)	0.999 (0.00)	0.997 (0.00)	1.00 (0.00)	0.999 (0.00)	0.998 (0.00)
3	0.05	20000	***r****	1.00 (0.00)	0.999 (0.00)	0.997 (0.00)	1.00 (0.00)	0.998 (0.00)	0.996 (0.00)
4	0.05	20000	***r****	1.00 (0.00)	0.997 (0.00)	0.997 (0.00)	1.00 (0.00)	0.999 (0.00)	0.998 (0.00)
5	0.05	20000	***r****	1.00 (0.00)	0.999 (0.00)	0.998 (0.00)	1.00 (0.00)	0.999 (0.00)	0.998 (0.00)
1	0.05	10000	***r****	0.999 (0.00)	0.994 (0.00)	0.985 (0.00)	0.999 (0.00)	0.995 (0.00)	0.988 (0.00)
2	0.05	10000	***r****	1.00 (0.00)	0.997 (0.00)	0.993 (0.00)	1.00 (0.00)	0.998 (0.00)	0.996 (0.00)

3	0.05	10000	***r ****	1.00 (0.00)	0.998 (0.00)	0.994 (0.00)	1.00 (0.00)	0.996 (0.00)	0.991 (0.00)
4	0.05	10000	***r ****	1.00 (0.00)	0.998 (0.00)	0.994 (0.00)	1.00 (0.00)	0.998 (0.00)	0.995 (0.00)
5	0.05	10000	***r ****	1.00 (0.00)	0.998 (0.00)	0.995 (0.00)	1.00 (0.00)	0.999 (0.00)	0.996 (0.00)
1	0.05	3000	***r ****	0.997 (0.00)	0.982 (0.00)	0.955 (0.00)	0.998 (0.00)	0.985 (0.00)	0.963 (0.00)
2	0.05	3000	***r ****	0.999 (0.00)	0.992 (0.00)	0.979 (0.00)	0.999 (0.00)	0.995 (0.00)	0.986 (0.00)
3	0.05	3000	***r ****	0.999 (0.00)	0.992 (0.00)	0.980 (0.00)	0.998 (0.00)	0.989 (0.00)	0.971 (0.00)
4	0.05	3000	***r ****	0.999 (0.00)	0.993 (0.00)	0.981 (0.00)	0.999 (0.00)	0.994 (0.00)	0.985 (0.00)
5	0.05	3000	***r ****	0.999 (0.00)	0.994 (0.00)	0.985 (0.00)	0.999 (0.00)	0.996 (0.00)	0.987 (0.00)
1	0.05	500	***r ****	0.957 (0.03)	0.901 (0.03)	0.813 (0.02)	0.963 (0.03)	0.936 (0.00)	0.829 (0.02)
2	0.05	500	***r ****	0.994 (0.00)	0.958 (0.01)	0.913 (0.01)	0.995 (0.00)	0.971 (0.00)	0.927 (0.00)
3	0.05	500	***r ****	0.993 (0.00)	0.961 (0.00)	0.905 (0.00)	0.992 (0.00)	0.952 (0.00)	0.883 (0.01)
4	0.05	500	***r ****	0.994 (0.00)	0.963 (0.00)	0.914 (0.00)	0.995 (0.00)	0.968 (0.00)	0.923 (0.00)
5	0.05	500	***r ****	0.995 (0.00)	0.973 (0.00)	0.928 (0.01)	0.995 (0.00)	0.974 (0.00)	0.936 (0.00)
1	0.5	20000	***CCC *****	0.999 (0.00)	0.979 (0.00)	0.921 (0.00)	0.999 (0.00)	0.979 (0.00)	0.922 (0.00)
2	0.5	20000	***CCC *****	0.999 (0.00)	0.980 (0.00)	0.923 (0.00)	0.999 (0.00)	0.980 (0.00)	0.922 (0.00)
3	0.5	20000	***CCC *****	0.999 (0.00)	0.980 (0.00)	0.922 (0.00)	0.999 (0.00)	0.979 (0.00)	0.918 (0.00)
4	0.5	20000	***CCC *****	0.999 (0.00)	0.979 (0.00)	0.920 (0.00)	0.999 (0.00)	0.977 (0.00)	0.913 (0.00)
5	0.5	20000	***CCC *****	0.999 (0.00)	0.978 (0.00)	0.918 (0.00)	0.999 (0.00)	0.976 (0.00)	0.910 (0.00)
1	0.5	10000	***CCC *****	0.998 (0.00)	0.977 (0.00)	0.917 (0.00)	0.999 (0.00)	0.978 (0.00)	0.919 (0.00)
2	0.5	10000	***CCC *****	0.999 (0.00)	0.979 (0.00)	0.922(0.00)	0.999 (0.00)	0.979 (0.00)	0.921 (0.00)
3	0.5	10000	***CCC *****	0.999 (0.00)	0.978 (0.00)	0.921 (0.00)	0.999 (0.00)	0.978 (0.00)	0.918 (0.00)
4	0.5	10000	***CCC *****	0.999 (0.00)	0.978 (0.00)	0.918 (0.00)	0.999 (0.00)	0.976 (0.00)	0.913 (0.00)
5	0.5	10000	***CCC *****	0.999 (0.00)	0.977 (0.00)	0.916 (0.00)	0.999 (0.00)	0.975 (0.00)	0.909 (0.00)
1	0.5	3000	***CCC *****	0.996 (0.00)	0.967 (0.00)	0.898 (0.00)	0.997 (0.00)	0.970 (0.00)	0.904 (0.00)
2	0.5	3000	***CCC *****	0.998 (0.00)	0.976 (0.00)	0.915 (0.00)	0.998 (0.00)	0.975 (0.00)	0.913 (0.00)
3	0.5	3000	***CCC *****	0.998 (0.00)	0.974 (0.00)	0.911 (0.00)	0.998 (0.00)	0.973 (0.00)	0.909 (0.00)
4	0.5	3000	***CCC *****	0.998 (0.00)	0.974 (0.00)	0.909 (0.00)	0.998 (0.00)	0.972 (0.00)	0.904 (0.00)
5	0.5	3000	***CCC *****	0.998 (0.00)	0.973 (0.00)	0.909 (0.00)	0.998 (0.00)	0.971 (0.00)	0.899 (0.00)
1	0.5	500	***CCC *****	0.987 (0.00)	0.921 (0.00)	0.819 (0.00)	0.965 (0.02)	0.931 (0.00)	0.816 (0.02)
2	0.5	500	***CCC *****	0.994 (0.00)	0.956 (0.00)	0.879 (0.00)	0.994 (0.00)	0.955 (0.00)	0.877 (0.00)
3	0.5	500	***CCC *****	0.993 (0.00)	0.950 (0.00)	0.870 (0.00)	0.993 (0.00)	0.950 (0.00)	0.863 (0.00)

4	0.5	500	***CCC* ****	0.992 (0.00)	0.946 (0.00)	0.861 (0.00)	0.993 (0.00)	0.948 (0.00)	0.859 (0.00)
5	0.5	500	***CCC* ****	0.993 (0.00)	0.951 (0.00)	0.866 (0.00)	0.992 (0.00)	0.942 (0.00)	0.847 (0.00)
1	0.3	20000	***CCC* ****	0.999 (0.00)	0.979 (0.00)	0.921 (0.00)	0.999 (0.00)	0.979 (0.00)	0.922 (0.00)
2	0.3	20000	***CCC* ****	0.999 (0.00)	0.979 (0.00)	0.921 (0.00)	0.999 (0.00)	0.978 (0.00)	0.919 (0.00)
3	0.3	20000	***CCC* ****	0.999 (0.00)	0.980 (0.00)	0.925 (0.00)	0.999 (0.00)	0.980 (0.00)	0.923 (0.00)
4	0.3	20000	***CCC* ****	0.999 (0.00)	0.978 (0.00)	0.918 (0.00)	0.999 (0.00)	0.979 (0.00)	0.942 (0.01)
5	0.3	20000	***CCC* ****	0.999 (0.00)	0.980 (0.00)	0.924 (0.00)	0.999 (0.00)	0.978 (0.00)	0.916 (0.00)
1	0.3	10000	***CCC* ****	0.998 (0.00)	0.976 (0.00)	0.917 (0.00)	0.999 (0.00)	0.977 (0.00)	0.919 (0.00)
2	0.3	10000	***CCC* ****	0.999 (0.00)	0.978 (0.00)	0.918 (0.00)	0.999 (0.00)	0.978 (0.00)	0.917 (0.00)
3	0.3	10000	***CCC* ****	0.999 (0.00)	0.979 (0.00)	0.923 (0.00)	0.999 (0.00)	0.979 (0.00)	0.922 (0.00)
4	0.3	10000	***CCC* ****	0.999 (0.00)	0.977 (0.00)	0.915 (0.00)	0.999 (0.00)	0.978 (0.00)	0.917 (0.00)
5	0.3	10000	***CCC* ****	0.999 (0.00)	0.979 (0.00)	0.922 (0.00)	0.999 (0.00)	0.977 (0.00)	0.915 (0.00)
1	0.3	3000	***CCC* ****	0.996 (0.00)	0.967 (0.00)	0.898 (0.00)	0.997 (0.00)	0.970 (0.00)	0.905 (0.00)
2	0.3	3000	***CCC* ****	0.998 (0.00)	0.974 (0.00)	0.910 (0.00)	0.998 (0.00)	0.973 (0.00)	0.908 (0.00)
3	0.3	3000	***CCC* ****	0.998 (0.00)	0.976 (0.00)	0.915 (0.00)	0.998 (0.00)	0.975 (0.00)	0.914 (0.00)
4	0.3	3000	***CCC* ****	0.997 (0.00)	0.972 (0.00)	0.904 (0.00)	0.998 (0.00)	0.974 (0.0)	0.910 (0.00)
5	0.3	3000	***CCC* ****	0.998 (0.00)	0.975 (0.00)	0.913 (0.00)	0.998 (0.00)	0.974 (0.00)	0.908 (0.00)
1	0.3	500	***CCC* ****	0.986 (0.00)	0.921 (0.00)	0.817 (0.00)	0.963 (0.03)	0.931 (0.00)	0.812 (0.02)
2	0.3	500	***CCC* ****	0.994 (0.00)	0.951 (0.00)	0.870 (0.00)	0.993 (0.00)	0.946 (0.00)	0.864 (0.01)
3	0.3	500	***CCC* ****	0.993 (0.00)	0.950 (0.00)	0.869 (0.00)	0.993 (0.00)	0.951 (0.00)	0.867 (0.00)
4	0.3	500	***CCC* ****	0.991 (0.00)	0.937 (0.00)	0.844 (0.00)	0.994 (0.00)	0.954 (0.00)	0.871 (0.01)
5	0.3	500	***CCC* ****	0.993 (0.00)	0.949 (0.00)	0.865 (0.00)	0.994 (0.00)	0.954 (0.00)	0.871 (0.00)
1	0.05	20000	***CCC* ****	0.999 (0.00)	0.979 (0.00)	0.921 (0.00)	0.999 (0.00)	0.979 (0.00)	0.922 (0.00)
2	0.05	20000	***CCC* ****	0.999 (0.00)	0.980 (0.00)	0.924 (0.00)	0.999 (0.00)	0.980 (0.00)	0.923 (0.00)
3	0.05	20000	***CCC* ****	0.999 (0.00)	0.981 (0.00)	0.926 (0.00)	0.999 (0.00)	0.979 (0.00)	0.920 (0.00)
4	0.05	20000	***CCC* ****	0.999 (0.00)	0.982 (0.00)	0.925 (0.00)	0.999 (0.00)	0.982 (0.00)	0.930 (0.00)
5	0.05	20000	***CCC* ****	0.999 (0.00)	0.981 (0.00)	0.925 (0.00)	0.999 (0.00)	0.981 (0.00)	0.928 (0.00)
1	0.05	10000	***CCC* ****	0.998 (0.00)	0.977 (0.00)	0.917 (0.00)	0.999 (0.00)	0.977 (0.00)	0.918 (0.00)
2	0.05	10000	***CCC* ****	0.999 (0.00)	0.979 (0.00)	0.922 (0.00)	0.999 (0.00)	0.979 (0.00)	0.922 (0.00)
3	0.05	10000	***CCC* ****	0.999 (0.00)	0.981 (0.00)	0.926 (0.00)	0.999 (0.00)	0.977 (0.00)	0.918 (0.00)
4	0.05	10000	***CCC* ****	0.999 (0.00)	0.980 (0.00)	0.924 (0.00)	0.999 (0.00)	0.981 (0.00)	0.928 (0.00)
5	0.05	10000	***CCC* ****	0.999 (0.00)	0.980 (0.00)	0.924 (0.00)	0.999 (0.00)	0.981 (0.00)	0.927 (0.00)

1	0.05	3000	***CCC *****	0.996 (0.00)	0.966 (0.00)	0.898 (0.00)	0.997 (0.00)	0.969 (0.00)	0.904 (0.00)
2	0.05	3000	***CCC *****	0.998 (0.00)	0.975 (0.00)	0.913 (0.00)	0.998 (0.00)	0.976 (0.00)	0.916 (0.00)
3	0.05	3000	***CCC *****	0.998 (0.00)	0.976 (0.00)	0.917 (0.00)	0.997 (0.00)	0.972 (0.00)	0.906 (0.00)
4	0.05	3000	***CCC *****	0.998 (0.00)	0.976 (0.00)	0.918 (0.00)	0.998 (0.00)	0.978 (0.00)	0.922 (0.00)
5	0.05	3000	***CCC *****	0.998 (0.00)	0.977 (0.00)	0.920 (0.00)	0.999 (0.00)	0.978 (0.00)	0.921 (0.00)
1	0.05	500	***CCC *****	0.957 (0.03)	0.893 (0.03)	0.793 (0.02)	0.963 (0.03)	0.928 (0.00)	0.804 (0.02)
2	0.05	500	***CCC *****	0.993 (0.00)	0.949 (0.00)	0.872 (0.01)	0.994 (0.00)	0.957 (0.00)	0.878 (0.00)
3	0.05	500	***CCC *****	0.993 (0.00)	0.949 (0.00)	0.868 (0.00)	0.991 (0.00)	0.939 (0.00)	0.847 (0.01)
4	0.05	500	***CCC *****	0.993 (0.00)	0.952 (0.00)	0.873 (0.00)	0.994 (0.00)	0.957 (0.00)	0.882 (0.00)
5	0.05	500	***CCC *****	0.994 (0.00)	0.958 (0.00)	0.883 (0.00)	0.995 (0.00)	0.960 (0.00)	0.892 (0.00)

* ***r:*** Pearson correlation

** **CCC**: Lin’s concordance correlation coefficient
